# Parenting Characteristics among Adults With Social Anxiety and their Influence on Social Anxiety Development in Children: A Brief Integrative Review

**DOI:** 10.3389/fpsyt.2021.614318

**Published:** 2021-04-28

**Authors:** Katelyn M. Garcia, Corinne N. Carlton, John A. Richey

**Affiliations:** Clinical Science Program, Department of Psychology, Virginia Tech, Blacksburg, VA, United States

**Keywords:** social anxiety disorder, parenting, overcontrol, warmth, adolescence

## Abstract

The purposes of this brief integrative review are to identify and critically evaluate recent work in the area of parenting processes that are disproportionately observed among parents with social anxiety disorder (SAD) that may ultimately increase risk among offspring, and to further link these processes to specific targets for intervention. Accordingly, we first evaluate the relevance of specific parenting styles as they pertain to increased risk of developing SAD among offspring. Second, we link these parenting processes to observations of certain unfavorable consequences among socially anxious youth, such as low perceived autonomy and poorer social skills. Finally, in light of these consequences we extend our conclusions into potentially modifiable targets among parents with SAD, focusing on the enhancement of autonomy and facilitating offspring's normative period of transition into independence during adolescence. Overall, we conclude that parenting behaviors commonly observed among adults with SAD, such as overcontrol and low parental warmth, likely have a direct impact on the development of social anxiety symptoms among their children. However, these parenting behaviors are plausibly modifiable and therefore repurposing existing interventions for use among parents with SAD in conjunction with interventions with their offspring is likely to provide direct clinical benefit.

## Influence of Parenting Characteristics on Social Anxiety Development in Youth

According to DSM-5, Social Anxiety Disorder (SAD) is classified as a persistent and marked fear of one or more social or performance situations in which the individual is exposed to unfamiliar people or to possible scrutiny by others. Individuals with SAD fear that they will behave in a way that will be embarrassing and humiliating (1). Except for separation anxiety disorder, which tends to onset in early childhood, epidemiologic research has indicated that SAD onsets earlier than other anxiety disorders, with a mean age of onset situated during early adolescence, at roughly 13 years of age (2, 3). Among adolescents, prevalence rates for SAD are between 10 and 15% (4, 5), thus ranking it among the most common anxiety disorders during this developmental period (6). This unique pattern of onset suggests that there are distinct factors emerging during the early adolescent period that may catalyze the development of social anxiety (SA) symptomatology. Given the various factors that have been implicated in the development of SAD, the disorder likely arises from a complex interaction of environmental and internal factors. Specifically, Rapee and Spence's (7) proposed etiological model of SAD classifies risk factors into environmental (parent/child interaction, aversive social experiences and negative life events) and internal factors (genetics, temperament, cognitive factors, and social skills deficits), and these factors may also be linked to environmental conditions or interact with environmental conditions. Prior work has established a robust link between adolescent SA and parenting behaviors, such as parental overcontrol, which has been observed among adults with SAD (8). However, relatively little work has explored these parenting behaviors and the mechanisms by which they interact with youth anxiety to increase risk for SAD genesis during the adolescent period. Accordingly, the purposes of this brief integrative review are to (1) characterize the literature on parenting behaviors that are disproportionately observed among adults with SAD and (2) review the literature that links these variables to unfavorable consequences in socially anxious youth. We further extend these mechanistic accounts to (3) identify promising avenues for intervention by considering modifiable targets within parents of at-risk youth. This review is not systematic in nature, but provides integrative synthesis and theoretical commentary to advance work in this area. For the purposes of this review, a literature search was conducted in PubMed using the following terms: “parenting,” “social anxiety,” “parental overcontrol,” “parental warmth,” “transfer of threat,” “maternal social anxiety,” “peer victimization.” Original articles that were published in the English language were considered. Due to the brief nature of this review, articles that were non-empirical or primarily theoretical in nature were excluded from the review process. Full-text articles were initially read for inclusion and two contributing authors contributed to the screening of articles.

## Parenting Characteristics Linked to Social Anxiety among Adolescents

### Parental Overcontrol

The relationship between anxiety symptoms in youth and parental overcontrol has been appreciated for some time (9–16). Parental overcontrol has been defined as a pattern of consistent over-involvement, including takeover of responsibilities and shielding children from new and potentially stressful situations (17). Prior work has highlighted the relevance of parental overcontrol in the development and maintenance of anxiety in youth (18). However, the specific mechanisms by which this process evolves among parents with social anxiety remain incompletely understood. Work from Ballash et al. (19) provides some insight as to why parents with SAD may implement overcontrolling behaviors. They were among the first to suggest that this parenting strategy may be intended to instrumentally shield offspring from excessive threat. For example, socially anxious parents may respond to the threat of negative evaluation by making more decisions for the child, which in turn may lower self-efficacy among offspring (20, 21). Similarly, socially anxious parents may limit opportunities for socialization between their child and same-age peers, thus increasing their child's behavioral avoidance of feared social situations (22).

In particular, maternal overcontrol has been consistently considered as a risk factor for the development of SA symptoms in youth (23, 24). Specifically, higher maternal overcontrol occurring at 7 years of age, as evaluated by ratings of overcontrol derived from Rubin and Cheah (25) during parent-child interaction tasks, has been demonstrated to predict higher social anxiety symptoms and lifetime rates of SAD on the Screen for Child Anxiety Related Emotional Disorders [SCARED; (26)] during adolescence (27). Mothers may exhibit overcontrolling parenting behaviors by limiting opportunities for their offspring to explore difficult social situations and develop coping skills (28). Thus, it can be theorized that mothers with SAD may exhibit increased overcontrol in social situations that present as interpersonally difficult and anxiety provoking. Children of overcontrolling mothers display lower self-autonomy needed to develop perceived control over their environment (29), thereby increasing the likelihood of fear in social situations (22). Mothers with higher anxiety have been found to exhibit overcontrolling behaviors, as measured by the Behavioral Involvement Parenting Scale [BIPS; (30)], in situations in which offspring face stressful stimuli in order to reduce their own physiological reactivity (17, 31). Gender may play an important role, such that overcontrol, as measured on the Psychological Control Scale—Youth Self-Report [PSC-YSR; (32)], has also been found to mediate the relationship between maternal (but not paternal) anxious parenting during a social stressor and daughter's SA symptoms (24).

Although less thoroughly explored than maternal overcontrol, recent findings also indicate that paternal overcontrol may be related to adolescent anxiety (23), and social anxiety specifically (33). For example, recent meta-analytic results have indicated that paternal parenting styles may potentially be more related to broadly defined anxiety symptomatology in offspring as compared to maternal parenting styles (23). However, this effect may also be influenced by gender, as demonstrated by Van Zalk et al. (34), who reported that boys' SA symptoms were associated with higher perceived parental overcontrol whereas girls' SA symptoms were associated with higher paternal worry, using a 5-point response scale derived from Kerr and Stattin (35). Taken together, the broader body of work exploring the interface between parental overcontrol and SA symptoms suggests that tactics aimed at restricting offspring's access to anxiety-inducing situations may ironically produce the very consequences they were intended to prevent.

### Low Parental Warmth

Parental warmth refers to expression of positive emotion during reciprocal interactions with offspring, including verbal expressions of “endearment, praise, and smiles” (36). It has been previously demonstrated that regardless of maternal anxiety symptoms, mothers of anxious children display lower warmth (37). For example, Crosby Budinger et al. (36) found that parents with SAD demonstrate less warmth and positive affect during parent-child performance tasks [coding system adopted from (38)], and moreover, early work has shown that adults with SAD retrospectively report that their own parents demonstrated less warmth (39, 40). Other studies have further suggested that in general, adults with SAD are less emotionally expressive (41, 42). For example, Davila and Beck (43) found that social anxiety symptoms were associated with specific interpersonal styles of avoidance, specifically avoidance of expressing strong emotions. Kashdan and Steger (44) theorized that socially anxious individuals adopt a less emotionally expressive strategy to lessen the likelihood that they will make a social error, which consequently also constrains prosocial behaviors as well (e.g., eye contact, smiling). This pattern of general emotional suppression among adults with SAD has been previously alleged to result in a parenting style that is lower in warmth (45).

“Cold” parenting styles appear to have especially poor implications for adolescents either with, or at risk for social anxiety (46). Specifically, Kaufman et al. (47) recently reported data indicating that low parental warmth, as measured by the Egna Minnen Beträffande Uppfostran Warmth and Rejection Scale [EMBU; (48)], predicted increases in peer victimization among offspring, and further that the relationship between children's victimization by peers and parent's warmth was mediated by children's social anxiety and depressive symptoms. These effects also appear to be relevant to later stages of development, as reported by Spokas and Heimberg (46), who illustrated that low parental warmth, as measured by the EMBU, also predicted increases in SA symptomatology during the first semester of college. Moreover, it has been demonstrated that a “cold” parenting style increases child risk for experiencing higher perceived interpersonal threat (49). Children of parents with colder parenting styles, such as those found in parents with SAD, may have a less explicit social referencing benchmark to follow, resulting in lower prosocial behavior (50). Consistent with this notion, Padilla-Walker et al. (51) demonstrated that children's familial prosocial behavior was associated with maternal warmth, and prosocial behavior with friends was linked to paternal warmth, as measured by the Parenting Styles and Dimensions Questionnaire [PDSQ; (52)]. Thus, one possible explanation for the relationship between low parental warmth and adolescent SAD development may therefore involve constricted emotional repertoire found among parents with social anxiety symptomatology, culminating in a parenting style that has been previously described as “cold.”

### Transfer of Threat Information

Parental modeling of social concerns (e.g., fearful behavior toward strangers) has been theorized to increase risk for offspring's SA symptoms in part through a process of observational learning (53). Such transmission of social threat may take place as children learn to rationally adjust their expectations about the environment based on observations of parental anxious talk or behavior. For instance, parents with SAD have been found to attribute more negative features to the environment, such as labeling certain environments as highly threatening (54, 55), which may increase vulnerability to the child and promote avoidance (56). Parents with social anxiety may also express potential threat through parent-child conversations that include parental catastrophizing comments (37), infrequent display of confident behaviors in specific environments (57), and promotion of child avoidance and lower encouragement to approach social situations (58). For example, during a task where mothers created a narrative about starting school, Murray et al. (55) demonstrated that socially anxious mothers displayed more negative (e.g., higher threat attribution) and less supportive (e.g., lower encouragement) narratives compared to controls, which was associated with childhood SA symptoms. In addition, high expression of parental anxiety in parents with lifetime SAD during early stages of childhood predicts increased fear and avoidance in children over time (59). In sum, children learn to form worries and threats about their environment by observing parents' own expressions of anxious thoughts or behaviors (57, 60). Therefore, lower maternal encouragement directed at the child to engage in potentially challenging social situations coupled with maternal negative representations of the child's environment may increase the child's anxious cognitions of potential threat and promote learning of avoidance strategies (55, 61, 62).

## Consequences of Unfavorable Parenting Characteristics

### Peer Victimization

Within the context of this review, overcontrol has been discussed as a characteristic of socially anxious parenting that may emerge to limit the child's exploration of difficult social situations. Here we discuss the relevance of overcontrol within general parenting populations on the consequences of peer victimization in youth in order to further demonstrate the effects this style of parenting may have on the child's social functioning. Increased sensitivity to rejection by peers and loneliness among youth have been previously associated with higher levels of parental rejection and overcontrol (63). Parental overcontrol, displayed by high parental intrusive demandingness, is also associated with heightened peer victimization among youth (64, 65). Specifically, parent-child relationships characterized by intense closeness, as found in overcontrolling relationships, are associated with higher levels of peer victimization in boys (64). Youth who reported greater peer victimization had an increased likelihood of developing an anxiety disorder and endorsed more severe anxiety symptoms 12 months later, with this link being strongest among children with parents high in anxiety (66). Thus, we postulate that peer victimization could potentially act as a mediating mechanism between parental overcontrol and the development of social anxiety symptoms in youth.

Higher levels of social anxiety in youth have been linked to specific, recurring peer victimization experiences, such as traditional bullying as well as cyberbullying (67). Furthermore, early retrospective studies on adults with SAD revealed self-reported childhood victimization including ridicule and teasing, which led to social avoidance or isolation during youth (68). Festa and Ginsburg (15) highlight that the strongest predictors of child self-reported social anxiety in youth ages 7–12 were parental overcontrol and social acceptance from peers. Furthermore, they note that parental rejection may promote negative self-evaluation in children (e.g., “something is wrong with me”), leading to feared peer rejection and increased social isolation. Regarding parenting behaviors, Perry et al. (69) found that overcontrolling parenting at age 2 was negatively associated with emotion regulation and inhibitory control by age 5. At age 10, these consequences were associated with more child-reported school and emotional problems, and less social skills and academic productivity reported by the teacher (69). Furthermore, children who do not experience positive peer relationships or good friendship quality (e.g., validation and support) may worry more about criticism and humiliation, thus increasing avoidance of social situations (15). Social support from friends and classmates (e.g., sharing problems, supporting ideas, telling them they are good at things, and caring about feelings) has been associated with less feelings of social anxiety, as measured using the Social Anxiety Scale for Adolescents (SAS-A) (70).

Parenting and peer victimization may interact to increase social anxiety symptoms in youth. In light of this framework, prior work has also discussed the importance of other childhood pathologies, such as attention-deficit/hyperactivity disorder (ADHD) in children with SAD and its relation to parenting and peer victimology. Specifically, Tükel et al. (71) have proposed a developmental theory that SAD may develop secondary to ADHD, such that children with ADHD display maladaptive social communication (e.g., forgetfulness, impulsive speaking) that is criticized by their parents and classroom peers; thus increasing feelings of humiliation and potential bullying, resulting in lowered self-confidence and fear of criticism. The child's social inhibition increases, thus resulting in social withdrawal.

### Lowered Autonomy and Self-Efficacy

An additional consequence of parental overcontrol is the restriction of the child's autonomy, encouraging children to become dependent or avoidant, specifically in social situations. In turn, this may hinder children from practicing appropriate social behaviors when faced with novel situations (34). For example, in a study where mothers and their offspring planned a speech together for the child to read aloud, Becker and Ginsburg (14) found that children whose mothers restricted autonomy by telling the child what to say reported higher levels of distress and had a poorer speech delivery as compared to children with mothers who displayed more autonomy granting. Although it is important to consider the level of autonomy restriction the parent may be placing on the child, prior literature has identified the child's perceived control over their environment as a potential additional factor (72). Moreover, Chorpita and Barlow (20) reported that lower perceived control makes children more vulnerable to environmental stressors and therefore may produce unwanted emotional outcomes (e.g., anxious behavior). In support of this framework, recent research has found that the association between autonomy granting and child emotional reactiveness was moderated by child perceived control in children with GAD, separation anxiety, and SAD, such that low autonomy granting combined with low child perceived control predicted poorer emotional outcomes (73). These results highlight the importance of the adolescent developmental stage, in which desire for increased control and autonomy normally increases, and directly competes with overcontrolling parenting styles that limit autonomous behaviors.

## Targets for Intervention: Opportunities to Modify Parenting Practices among Socially Anxious Adults

### Targeting Parenting Behaviors and Anxiety

Due to the presumed positive relationship between parental anxiety and overcontrolling behaviors, targeting parental anxiety using existing practices such as cognitive behavioral therapy (CBT) may prove useful in simultaneously decreasing anxiety as well as parental overcontrol. The rationale for such an approach is derived from previous findings that individuals with social anxiety demonstrate heightened fear of social threat in the environment (74, 75). Most available CBT-based approaches for adult anxiety often do not include parenting components; therefore, the establishment of cognitive behavioral tactics for parenting among adults with SAD should target overcontrol in particular, using such evidence-based practices as exposure (*in vivo* or imaginal) where parents slowly reduce control over their child over time. In a similar sense, including parent education training into CBT may also prove effective for reducing parental anxiety and overcontrol. One such treatment found promising results post-implementation of an early intervention program for preschoolers at high risk of developing anxiety disorders using a combined method of parent education sessions and social skills training for the children (76). The parent education portion utilized the “Cool Little Kids” program (77), which focuses on parenting management skills, such as reducing overprotective parenting behaviors, anxiety management, and learning *in vivo* exposure strategies for their child. Results showed that relative to waitlist controls at the 6-month follow-up, children showed significantly fewer symptoms attributable to anxiety disorders and maternal anxiety symptoms decreased. Of note, children who did not receive the social skills training but had parents who received the parent education components also saw reductions in child anxiety symptoms, thus highlighting the utility of parent education alone in reducing the child's anxiety.

As noted earlier, parents with SAD have been found to demonstrate low warmth toward their offspring (36), which may potentiate anxiety symptoms. Therefore, treatment programs promoting parental warmth may prove particularly effective. In support of this notion, “Tuning in to Kids” [TIK; (78)] has been proposed as an early intervention parent training program that improves emotional socialization practices (e.g., regulation, awareness, emotion coaching) in parents with preschool aged children. Parents participating in TIK for six sessions reported increased emotional awareness and regulation, increased emotional coaching of their child, and decreases in emotionally dismissive behaviors (78). Similarly, in a study using TIK for Dads (“Dads Tuning in to Kids”), fathers reported a greater use of expressive encouragement and empathy, and increased prosocial skills in their children (79). Thus, adaptations of these parent training approaches have the potential to act as a protective factor against youth social avoidance and anxiety by increasing prosocial behaviors in youth via parent's use of empathy and encouragement.

### Targeting Children's Anxiety and Autonomy

This brief integrative review has highlighted the importance of granting autonomy to youth and that specific parenting behaviors observed within SAD samples may restrict such autonomy. Perceived competence has been found to moderate the relationship between low autonomy granting and child emotional reactivity, and children who perceive high self-control over adverse life events report less anxious reactivity and more use of emotion regulation skills, such as problem solving (73). It therefore follows that increasing autonomy among at-risk youth may prove particularly effective in preventing or ameliorating symptoms of SA. In support of this notion, family cognitive behavioral therapy [FCBT; (80)], has been found to increase autonomy granting parenting and decrease overprotective parenting (81), which may have significant promise for use among parents with SAD. Of note, previous research on the efficacy of FCBT on decreasing child anxiety found that adolescents, 13 years and older, improved less on anxiety symptomatology as compared to younger children ages 8–12 (82), therefore providing further support for early intervention. In addition to FCBT, children with ADHD and comorbid SAD may wish to pursue treatments targeted at increasing executive functioning, such as “Unstuck and on Target” (83). Incorporating such treatment methods may increase social communication skills, thus decreasing feelings of inadequacy and promoting an increased sense of self-control.

### Increasing Child Socialization and Decreasing Parent Transfer of Threat

Parents with SA may transfer threat of the environment through parent-child conversations that include parental catastrophizing comments (37). Although a parent or child can receive individual treatment to decrease social anxiety regarding novel situations and environments, family treatment including aspects of socialization for both parent and child may also be effective. Specifically, high levels of social support and acceptance from peers have been linked to lower levels of social anxiety symptoms (15), thus supporting the need for incorporating increased socialization and social skills into treatment for both the parent and offspring. Given the importance of friendship quality and peer relationships with classmates (70), parents should encourage socialization both in and outside of the classroom that focuses on support and validation of ideas and feelings between youth. Barlow and Seidner (84) evaluated parental involvement in CBT for agoraphobia in adolescents, specifically in role-playing or exposure-based tasks. It was demonstrated that during exposure tasks, adolescents frequently asked their parents for assistance when managing emerging anxiety symptoms. Parental reaction ranged from “directive” responses (e.g., directly guiding the exposure tasks) to “supportive” (e.g., more autonomy granting). Considering the potential benefits of autonomy granting, encouraging parents with SAD to take a more supportive role in their child's exposures to feared social situations may therefore lessen transfer of threat information by allowing independent problem-solving and coping skills.

## Conclusions

Parents with SAD have been found to implement specific parenting behaviors that are associated with the development of social anxiety symptoms among their offspring. These parenting behaviors, which include overcontrol, low warmth, and transfer of threat information, have been further associated with unfavorable outcomes in offspring such as increased peer victimization, lower autonomy and emotional problems. Research regarding specific parenting characteristics of parents with SAD is limited in nature, and most of this work has focused on the maternal characteristics, whereas fathers with socially anxious symptoms are less commonly discussed. Further complicating the issue, there are inconsistent findings among the literature regarding maternal vs. paternal parenting styles, such that the majority of the literature suggests that maternal parenting styles are more related to offspring anxiety, whereas a separate body of work suggests that paternal parenting styles are instead more strongly related to child anxiety symptoms. Thus, there remain some discrepant findings that must be addressed in order for the field to effectively proceed. Limitations of the field further include scarcity of controls as comparison groups, such as parents without anxiety symptoms. Some of the current empirical literature has compared parents with social anxiety to parents with other anxiety disorders, such as generalized anxiety or agoraphobia, which limits conclusions regarding anxious vs. non-anxious parenting behaviors and determining normalcy of parenting behaviors. Another limitation to note is lack of standardization among observational tasks to measure overcontrolling behaviors in a laboratory setting, and further that coding schemes within these tasks differ significantly across research groups.

Limitations of the current review should thus be considered in light of these general limitations inherent in the literature. More specifically, this integrative review explored overcontrol, transfer of threat, and low parental warmth as specific parenting characteristics among parents with SAD, however discussions comparing these results to control groups or other anxiety disorders is limited due to the nature of the existing literature. Furthermore, this review focused primarily on adolescence and therefore discussion on the influence of parenting characteristics of parents with SAD may not be generalizable to other developmental periods. Lastly, this review is not systematic and therefore did not include all empirical findings.

We propose that possible modification of parenting behaviors, specifically overcontrol and low warmth, through the use of parent education training may likely prove beneficial for protecting against the development of social anxiety symptoms in offspring. In addition to treatment of social anxiety symptoms in youth combined with parenting therapies, incorporating aspects of increased autonomy and socialization may promote coping and social skills necessary for positive social interaction that otherwise is diminished from overcontrolling parenting. In light of the findings outlined here, future research may wish to consider the direct causal effects of modifying overcontrol, low warmth, and transfer of threat on the adolescent's perceived self-control and efficacy in relation to social anxiety symptomology. Likewise, treatment studies for adolescent social anxiety should explore the dynamics of parenting styles and youth reported symptoms.

## Author Contributions

KG, CC, and JR were involved in the conception, drafting, and revisions of the manuscript. All authors contributed to the article and approved the submitted version.

## Conflict of Interest

The authors declare that the research was conducted in the absence of any commercial or financial relationships that could be construed as a potential conflict of interest.
